# Annotations

**Published:** 1892-06-11

**Authors:** 


### ANNOTATIONS.

**Mirth and Medicine.**

"A MERRY heart doeth good like a medicine," said Solomon. Was that merely a little bit of flamboyant talk, or was it a definite, scientific, and demonstrable proposition and truth? A merry heart does a great deal more good than some medicines, than most medicines, perhaps. It sends the wholesome blood dancing through the brain and makes the world rosy and delightful. There is no appetiser like a merry heart, and as for digestion, the merriest have ever the best. What a splendid worker it is too! "A merry heart goes all the day, your sad one tires in a mile, aye." But our's is not an age of merry hearts. Soured hearts, disappointed hearts, jealous hearts, splenetic hearts are as plentiful on all hands as spring prickles after a "black-thorn winter." That is not a hopeful state of domestic business. What is it that makes us English so preternaturally sober, and our London English sober with a dash of bitterness? We are not all rich and great; that is the tonic at which we make such wry faces. So many of us have enough but nothing to spare; as much as our neighbours, but not sufficient to enable us to separate ourselves from them and assume an air of distinction all our own, that we feel life to be an exceedingly undiscriminating affair, and almost a swindle. There should be a cure for this sick egoism somewhere. The cure will perhaps be found in a careful study of some of our fellow-country-men who are rich enough in all conscience, and separate enough from their neighbours, though they are not, perhaps, so distinguished as they would like. Are these very rich ones so happy after all? The writer knows at least two millionaires well enough to speak on the subject of their happiness. They are both below the average in this respect. They could hardly be merry if they tried. One of them is by no means able to decide the knotty question whether or not life be really worth living. If we are to wait until we are distinguished before we will consent to be merry, some of us will need a fine stock of patience. It is not easy to be distinguished among thirty-five millions of people, and those people Englishmen. The old people, our national ancestors, won distinction, not by longing for it, and always aiming at it, but by doing their work from day to day magnificently, and by enjoying life as they went on. This is the way of happiness and mirth: work well done, and pleasure found in the doing and completing of it. As for fame, he who craves it most will enjoy it least when it comes.

**Scarlet Fever Treatment and Disinfection.**

MR. BRENDON CURGENVEN claims to cure scarlet fever patients, and to disinfect them so completely that they may safely run about and play with the other members of their families in seven to ten days from the inception of the attack. We have been accustomed to think that seven weeks was more nearly the period of safety. The new method is a great advance in an important department of medical practice, if it should prove in all hands to be as trustworthy as Mr. Curgenven thinks it is. It is very desirable that experiments be made on as large a scale as possible, both in fever hospitals and in private practice. With a view to the facilitation of these experiments we would urge upon readers to consider not only the substances and the methods employed by Mr. Curgenven, but also the rationale of the business; for no progress will ever be made unless we can get beyond the "rule of thumb." Shortly and simply, Mr. Curgenven uses Tucker's Eucalyptus Disinfectant, which consists of two or three essential oils of different densities, with thymol and camphor dissolved in the oil of Eucalyptus Globulas. The air of the sick room is saturated with the vapour of the disinfectant, and the body of the patient is smeared over with it night and morning for the the first three days, and after that once a day, following a warm bath, for seven days more. The case is then safe in all respects. This is the treatment in practice. The science which underlies it must also be understood. Here it is in a few words. Essential oils are very powerful bactericides; much more so than even strong solutions of carbolic acid. Their vapours—and they vaporise rapidly—are also powerful bactericides. As small a quantity as a half per cent. of the essential oil of cassia, cloves, cinnamon, eucalyptus, or cajuput will preserve milk, cream, paste, beef broth, or hay infusion from decomposition. Air fully charged with the vapour of many of the essential oils will destroy the bacilli of diphtheria, typhoid, tubercle, and anthrax, and it will preserve beef and hay infusions from the development of micro-organisms. If patients with scarlet fever inhale this bactericidal, but otherwise harmless, vapour, and have their skins anointed with the essential oils, they are treated completely within and without; and, Mr. Curgenven assures us, that the success is as complete as the treatment. The history and logic of this new departure can be obtained for a small sum in a little book entitled "The Disinfection of Scarlet Fever." The publisher is Mr. H. K. Lewis, of Gower Street. All fever hospitals should have the book, and all general practitioners and nurses.

**Town Children's Summer.**

ONE of the most depressing facts in the life of large towns is the almost complete lack of season, as seasons are understood and enjoyed in the country. In town the only conspicuous change which the average child appreciates is the change of temperature with the change of time. Town life, notwithstanding its supposed superior liveliness, is in this respect far more dull and monotonous than life in the country. The change from winter to spring is perhaps the most striking of all our seasonal changes. The peculiar pleasures of spring are all but unknown to the denizens of our streets and poorer squares. No man, one would think, can be an Englishman in the proper sense of the word who spends the whole of his life in the streets of towns. He may be a Manchester man or a Cockney, but not an Englishman. In order to know what it is to be a true "John Bull," he must have some familiarity with woods and fields, with cattle and farm. Some people probably think the smart town-bred workman or child very superior to the homely countryman of more old-fashioned times. We cannot agree with them. But even if it be granted that the countryman is less sprightly in his wit, it cannot be denied that he has far more knowledge of nature and the real world we live in, and far more breadth and practical capacity for dealing with a great variety of circumstances. If we are bent upon making all our men town men we must give them at least an occasional and temporary acquaintance with the country, its life, and its wild creatures, if only to let them see what the real earth is like, on the surface of which they have to live and die. The day in the country which used to be given to town children is being expanded into a few weeks. Apart from the pleasure and physiological profit of this to the children, it is to be looked upon as an important branch of education. A month in the country cannot but have a similar effect upon the narrow experience of town children to that produced upon young Englishmen of former generations by the making of "the grand tour." It is a new life and a new world which they temporarily share. We hope that truly wise and large-minded philanthropists will see the educational, as well as the physiological and happiness value of a few weeks in the country for town children, and will carry out all their plans on this larger and much more valuable scale.

